# The Open Birth Interval: A Resource for Reproductive Health Programs and Women's Empowerment

**DOI:** 10.9745/GHSP-D-19-00056

**Published:** 2019-09-23

**Authors:** John Ross, Kristin Bietsch

**Affiliations:** a Independent Consultant, New Paltz, NY, USA.; b Avenir Health, Glastonbury, CT, USA.

## Abstract

The open birth interval is the time since a woman's last birth. It reflects not only desire for contraception and child health services but also freedom for outside activities, employment, and personal autonomy. It merits attention from policy makers, program managers, and service providers.

## INTRODUCTION

Every woman who has ever given birth has a most recent birth, and she now stands at some point removed from it, in an “open interval.” Some women go on to another pregnancy and birth, but some never do, remaining permanently in the open interval. This interval is entirely different from the well-known “closed interval,” which pertains only to the time between 2 births in the past. The open interval can be determined from a simple question: “How long has it been since your last birth?” Recent national surveys can provide a current snapshot, and changes through time can be detected by reference to earlier surveys.

We undertook research on the open interval, with the expectation that it holds promise for a deeper understanding of reproductive behavior, women's status, and demographic processes. This study was exploratory in nature, rather than an investigation of a specific hypothesis. It required assembling the first general body of empirical information on interval lengths, and it included the following research questions:

- Currently, how are women distributed by the age of their youngest child?
- How does this distribution vary over time, by region, and by fertility levels?
- How does the open interval distribution relate to personal characteristics (age, wealth quintiles, etc.) and to reproductive health variables (contraceptive use, unmet need for contraception, intention to use a method)?
- How do these variables change within the first year after birth and in each ensuing year for the age of the youngest child?
- Has the declining percentage of women having children been offset by the overall increasing number of women, leading to a higher absolute number of women needing pregnancy and child care services?
- What are the likely policy and program applications of information on numbers and trends in the age of the youngest child?
- What are the implications of the age of the youngest child for women's roles, their freedom to pursue activities other than those related to pregnancy and child care, their participation in the labor force, and their empowerment?

In this commentary, we discuss our findings for these questions and show how the open interval distribution, based upon a single question in national surveys, can usefully augment other information for analytic and management purposes.

## LITERATURE REVIEW

Literature on the open interval is primarily composed of highly technical modeling work, starting in the mid-1960s, with publications by Sheps et al. in 1967^1^ and 1970^2^ and later by Srinivasan^3^ and Schmertmann,^4^^,^^5^ among others. Some of the literature conveyed skepticism about the usefulness of the open interval for program applications, but such doubts were expressed without the benefit of actual data for countries showing the interval's close relationship to contraceptive use, unmet need, and fertility measures. A recent review by Singh^6^ provides a thorough summary of the modeling analyses through 2015 and concisely explains their main assumptions. Some of this literature focuses on the possible relationships between closed and open intervals. Very little empirical information has been available on actual open intervals until now, when we have a large set of national surveys providing data over time on women's intervals since their last birth.

Very little empirical information has been available on actual open intervals until now.

However, for 4 countries—Ethiopia, Kenya, Tanzania, and Zimbabwe—a notable analysis of past survey trends incorporates both closed and open intervals, using technical methods to combine data to estimate both time trends and determinants.^7^ This analysis reveals that intervals in all 4 countries have lengthened, predominantly due to increased use of contraceptives. Intervals in these countries now range from 35 to 51 months. The intervals have been lengthening continuously, notably at a faster pace and to greater durations in urban areas compared with rural areas.

An early Taiwan survey in the 1960s that included the open interval showed that it explained socioeconomic variations in fertility levels at ages 30–39 better than past closed intervals. That survey further showed that contraceptive practice had helped extend the intervals.^8^

One experimental study included the open interval as a predictor of contraceptive adoption.^9^ It used before and after surveys in 2 Korean counties to test which baseline characteristics of the women would correspond to adoption of a method between the surveys. These characteristics included items such as age, family size, education, having enough sons, stated desire to use a method, and exposure to mass media, in addition to time since the last birth. In the county with a strong family planning program, 57% of women with open intervals shorter than 30 months adopted a method, but only 9% did so if the intervals were longer. In the other county, which had a weaker program, the stated desire to use a method was the strongest predictor.

One section below concerns the first year after birth, termed the “extended postpartum period, defined as the first year after birth.” There is an extensive literature on women's needs and behavior in that year, but here we refer to only selected sources, as explained in that section, for postpartum programs offered at or soon after women give birth.

## DATA AND METHODS

This study uses 232 Demographic and Health Surveys (DHS) conducted from 1985 onward in 74 countries, of which 56 have multiple surveys. The focus throughout is on the time since the woman's last birth, for currently married/in union women of reproductive age (15–44 or 15–49 depending on the survey). The analyses below pertain only to married women who have had at least 1 birth, although for brevity we refer simply to women. The inclusion of unmarried women would have complicated this first examination of open intervals across many countries. Countries are weighted equally in all averages presented below.

The focus throughout is on currently married/in union women of reproductive age and the time since the women's last birth.

The key DHS variable in our study is the time elapsed since the most recent birth, and we use this period as an approximation for the age of the woman's youngest child. In doing so, we neglect errors due to nonreporting of a most recent birth when the child died, in which case the youngest child is from an earlier birth. For example, if the latest birth was 10 years ago but the child died, the woman may be reporting a birth that occurred 12 years ago.

Pregnant women are usually included with the women closest to birth (those in the first 3 months or alternatively the first year after birth). Pregnant women represent 9% of all married women, on average, or 31% of those within the first-year category. The actual measurement of the open interval is subject to errors, as with misdating of births and with births omitted due to infant deaths; also, the data on current pregnancies omit early, unrecognized conceptions. We assume here that the error components are minor for our purposes, are constant for trend estimates, or both. Unmet need, referring basically to women not using any method but who are still fecund and wish to avoid pregnancy, has been defined technically with variations, but as used in this article, it follows the standard DHS definition. The various alternatives that have been suggested^10^ give both lower and higher estimates of unmet need. For this study, however, we use the DHS definition, as taken from the survey files. For some older surveys, we calculated unmet need using the Stata Do-file available on the DHS website. Actual open intervals can extend from a single birth when a woman is 15 years old to when she is age 50, for a maximum of 35 years, but in practice most are far shorter.

Stata 15 and R software were used to access the individual survey files and tabulate the data. The analytic methods included tabulations across countries and over time, with selected summary measures and regression techniques.

## RESULTS

### Patterns Among Countries and Over Time

We use 2 figures to illustrate first, the character of the open interval distribution, and second, to show how the distribution differs by country and over time. These figures use 1- or 5-year intervals up to 15 years (i.e., they include women whose youngest child is up to 15 years old).

**FIGURE 1:**
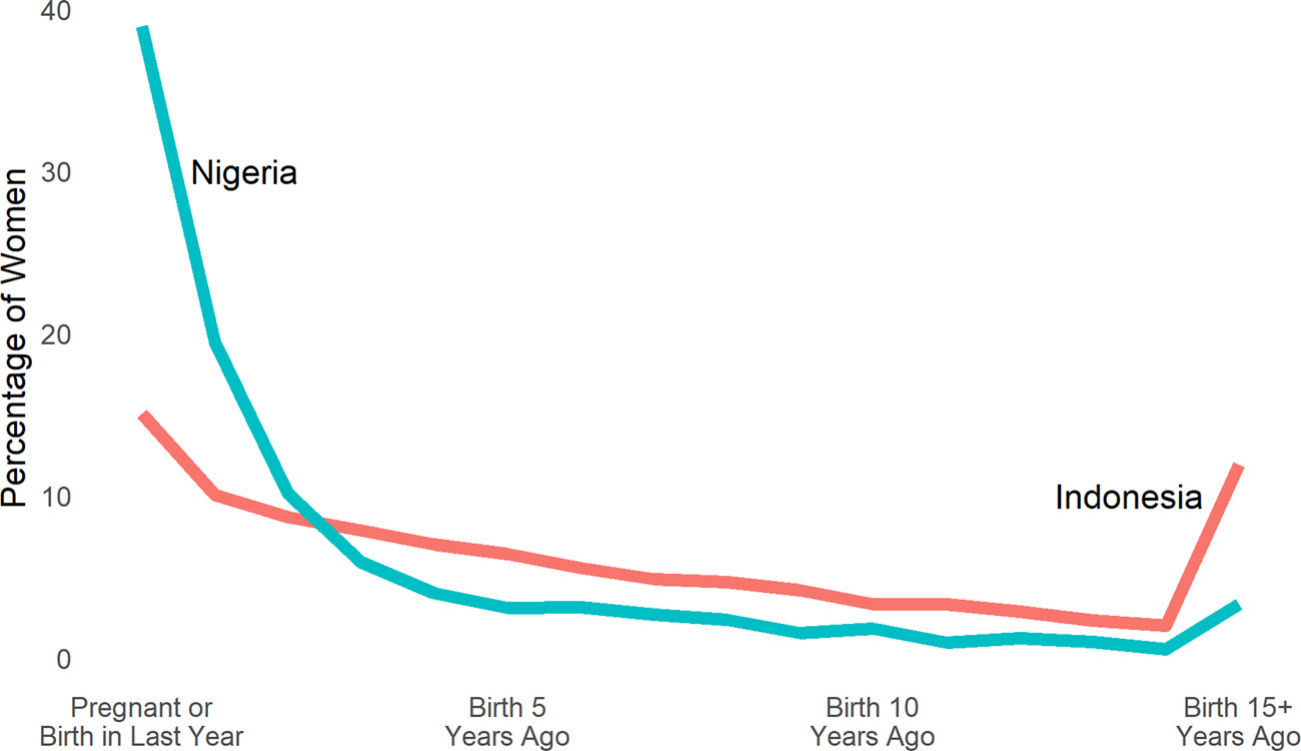
Distribution of Women of Reproductive Age by the Open Birth Interval in Nigeria and Indonesia ^a^ Based on latest Demographic and Health Surveys for Nigeria (2013) and Indonesia (2012).

In Figure 1, we compare Nigeria and Indonesia to illustrate the differences between a high-fertility country, with women clustered close to a recent birth, and a mid- to low-fertility country, with a broader spread. Women in Nigeria, a high-fertility country, are more heavily involved in childbearing compared with women in Indonesia, which has a lower fertility rate. Consequently, the resource demands on pregnancy, delivery, and early child care are considerably different between the 2 countries.

**FIGURE 2:**
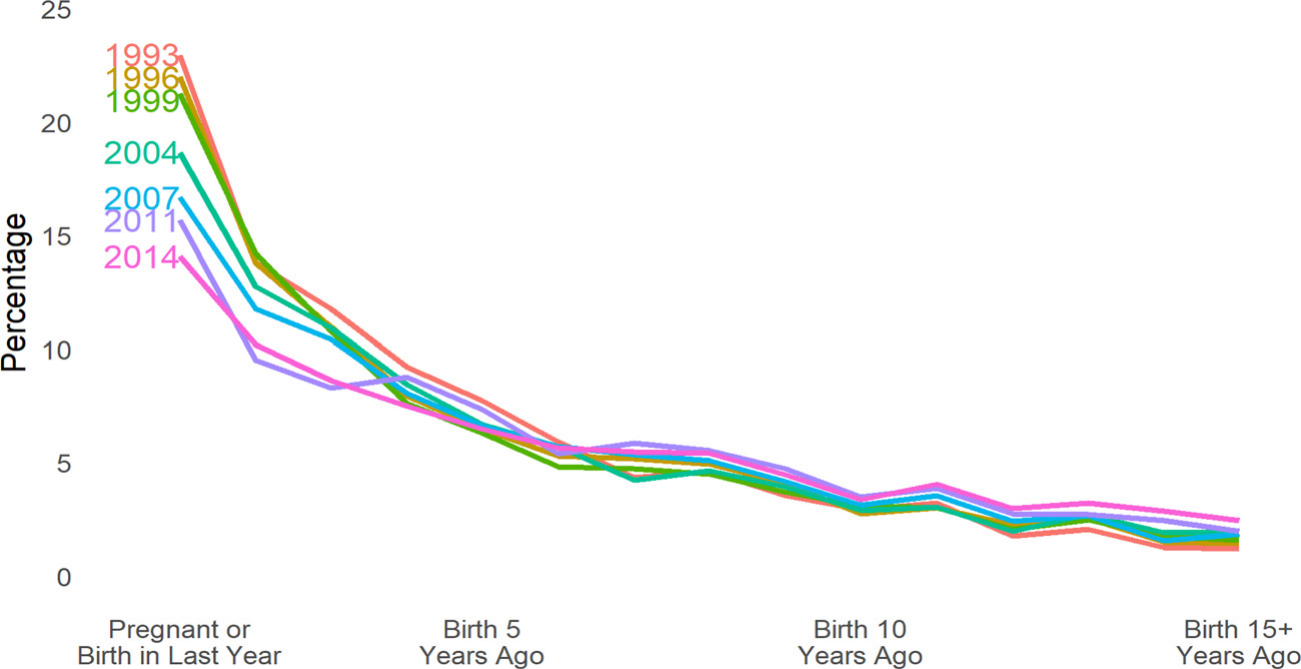
Distribution of Women of Reproductive Age by the Open Birth Interval in Bangladesh, Demographic and Health Surveys in 1993, 1996, 1999, 2004, 2007, 2011, and 2014

Further, such demands can change over time, as illustrated by the trends in Bangladesh (Figure 2). The percentage of women either pregnant or within a year of delivery has fallen from about 25% in 1993 to about 13% in 2014. Such a large reduction, of about half, has clear implications for changes in managerial decisions and for the health ministry's plans with regard to supplies, types of clinic loads, personnel decisions, and budgetary allocations. Only through the open interval distribution is information available to contrast the service needs of women with children at various ages.

Changes in open birth intervals have clear implications for planning with regard to supplies, types of clinic loads, personnel decisions, and budgets.

On average over all 74 countries, a remarkable one-fourth of women are pregnant or in the first year after birth and another fourth are clustered in their second or third year. The other half are spread thinly, at declining percentages, over the ensuing years, albeit with a peak of 8% in the final interval, which represents women whose youngest child is aged 15 or older (Table 1).

**TABLE 1.** Distribution of Women of Reproductive Age by Length of Open Interval, Latest Demographic and Health Surveys

Length of Open Interval in Years	Percentage
Pregnant/0–.9	25.6
1.0–1.9	14.8
2.0–2.9	10.1
3.0–3.9	7.2
4.0–4.9	5.6
5.0–5.9	4.6
6.0–6.9	4.5
7.0–7.9	3.7
8.0–8.9	3.2
9.0–9.9	2.7
10.0–10.9	2.6
11.0–11.9	2.1
12.0–12.9	2.0
13.0–13.9	1.8
14.0–14.9	1.5
15+	8.0
**Total**	**100.00**

Supplement 1 documents the variation around the average figures, by country and by region. Among regions, the proportion of women who are pregnant or have an infant is as low as 10% in Europe/West Asia and as high as 36% in West/Central sub-Saharan Africa. Among countries, the proportion is 15% or less in India, Bangladesh, Vietnam, Indonesia, and Nepal, as well as in Kazakhstan and Turkey, and in most countries listed in Europe/West Asia. The highest percentages are in the 2 sub-Saharan regions, exceeding 40% in Chad, the Democratic Republic of the Congo, and Niger. The range represented by the various countries—from 10% to over 40%—implies a great deal about the variation in the daily activities and personal options for the women involved, given how much women's lives can change when they no longer have the care of very young children.

**FIGURE 3:**
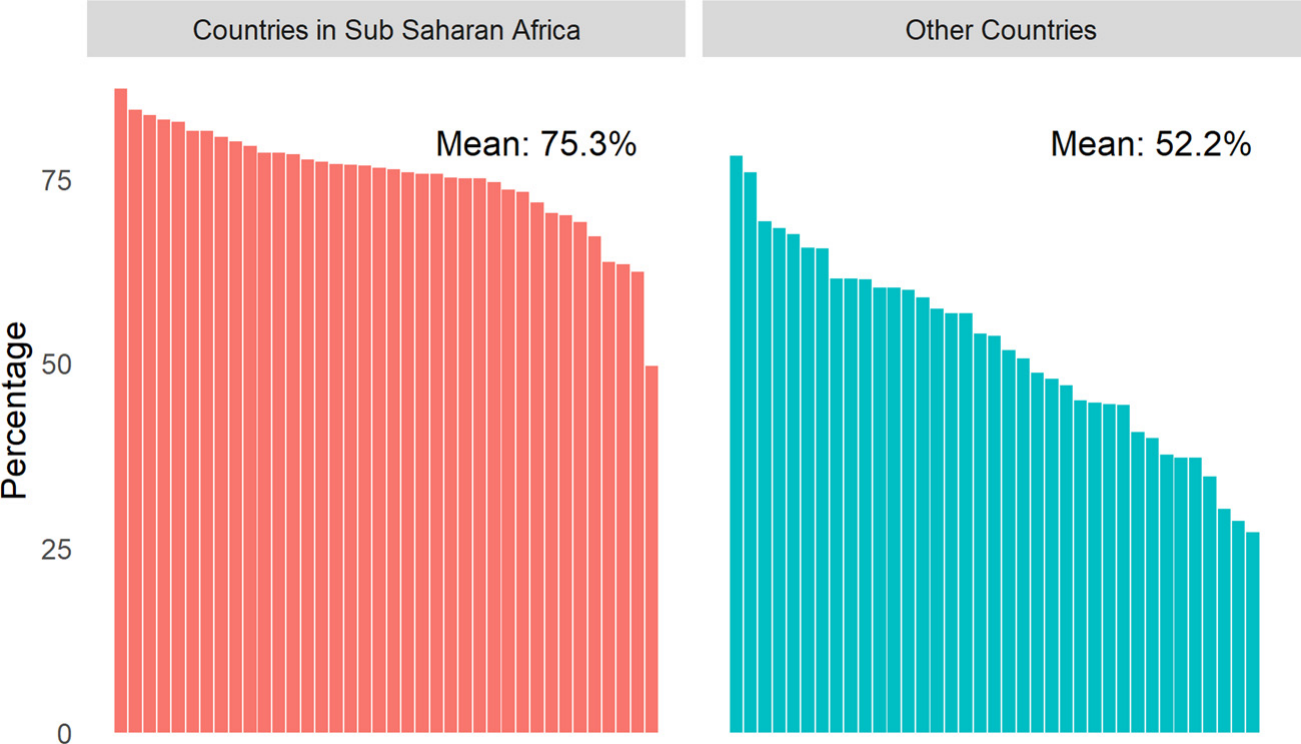
Percentage of Women With a Child Below Age 5 in Sub-Saharan Africa Compared to Other Regions: 74 Countries, Latest Demographic and Health Surveys, Various Dates

The greatest regional contrast is between sub-Saharan Africa (38 countries) and other regions (36 countries), as depicted in Figure 3, which displays the range of variation within each of the 2 regional groupings, as well as the dissimilar averages. In nearly all sub-Saharan African countries, more than 70% of women have a young child under age 5, while in the other regions the percentage is at or well below 60% in most countries. The percentages are much higher and closer together in sub-Saharan Africa than elsewhere; consequently it bears a much heavier burden of health and demographic investments for youth.

### Birth Intervals Are Lengthening in Most Countries

As contraceptive use has increased over the years, more women are going longer without a next birth. In our data set, 56 countries had multiple surveys, and a comparison of the earliest and latest survey in each country (average 17.3-year gap) shows a drop from 32.7% to 26.5% of women in the first interval (pregnant or in the first year after birth) and an increase from 26.1% to 31.3% in the final interval of over 5 years (data not shown). The intermediate intervals are consistent with that transition, with an initial decline and a subsequent increase. These changes modify the circumstances of many women as they are freed from the care of children in their early years of life. An additional view of the lengthening intervals over time in these countries is provided in the section labeled “A Simple Model Captures the Open Interval Distribution.”

### The Mix of Reproductive Statuses Evolves Over the Intervals

**FIGURE 4:**
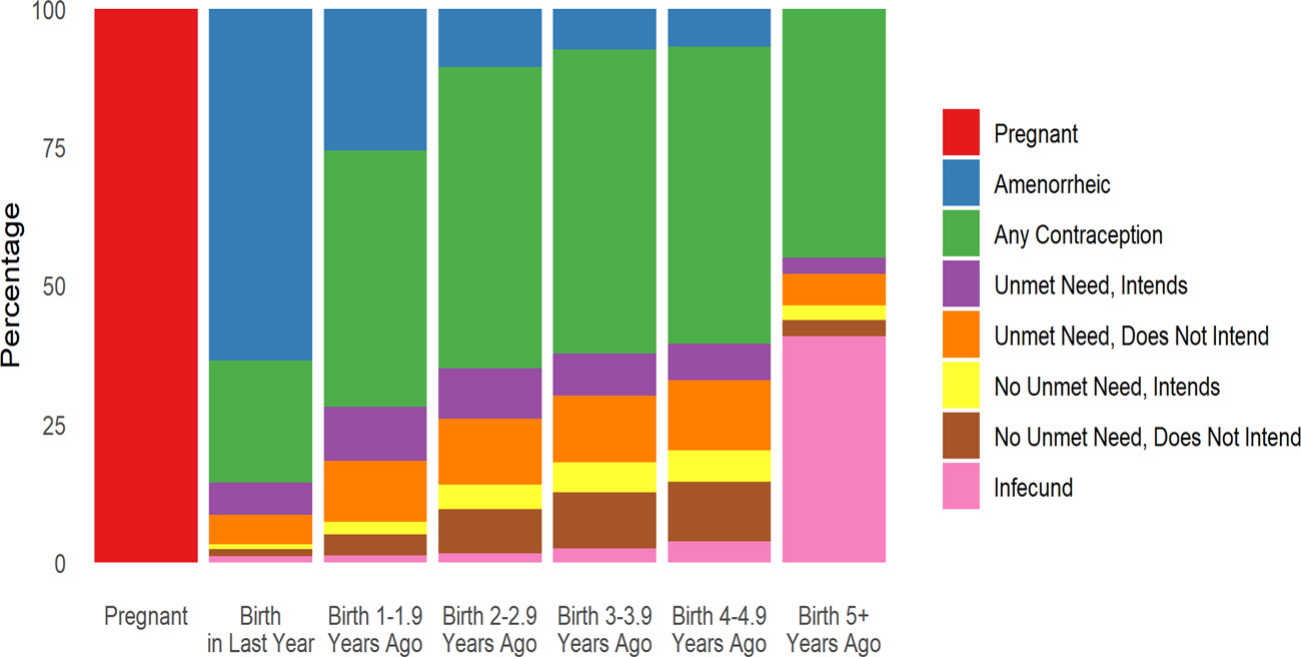
Distribution of Women by Open Birth Interval and Reproductive Health Status: 74 Countries, Latest Demographic and Health Surveys, Various Dates

**FIGURE 5:**
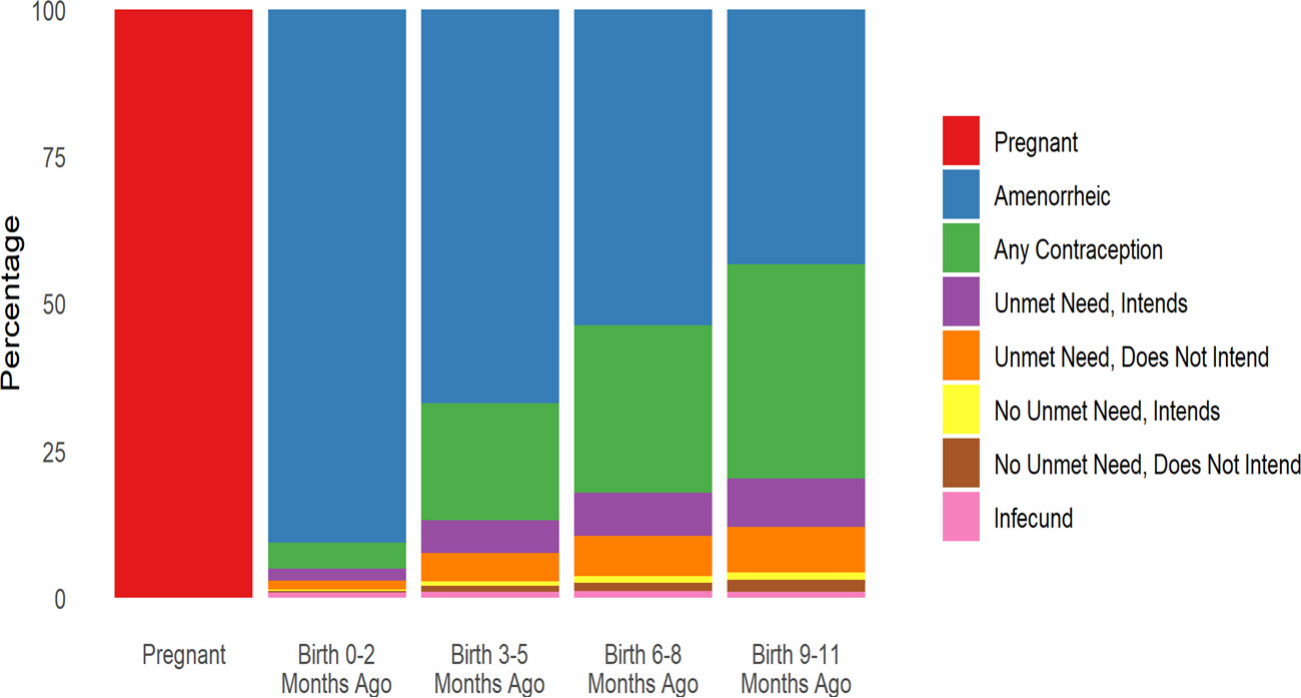
Distribution of Women by Open Birth Interval by Quarter Within the First Year After Birth, According to Reproductive Health Status: 74 Countries, Latest Demographic and Health Surveys, Various Dates

Rapid changes occur in women's lives as their youngest child ages. Figure 4 separates women in each interval after birth into mutually exclusive categories (adding to 100%) to show the major shifts. It is understood, however, that overlaps exist between categories. In particular, many pregnant women also have unmet need under the DHS definition, so Table 2 separates out all women with unmet need to clarify the proportion who are currently pregnant. (This approach also applies to Figure 5.) In Table 2 and in both figures, the intention to use contraception (“intends”) is recognized separately from unmet need.

**TABLE 2.** Profiles of Women of Reproductive Age by Contraceptive Use, Unmet Need, and Intention to Use a Method: Unweighted Averages for 74 Countries, Latest Demographic and Health Surveys, Various Dates

	Pregnant	Open Interval (Months)						Total
		0–11	12–23	24–35	36–47	48–59	60+	
**All women**	8.4	17.6	14.9	10.2	7.2	5.6	36.1	100.0
**Contraceptive use**								
None	15.8	20.5	12.1	7.2	5.2	4.1	35.1	100.0
Traditional		14.7	18.1	13.2	8.8	7.0	38.2	100.0
Modern, short-acting		16.8	20.9	15.5	10.6	7.4	28.8	100.0
Modern, long-acting		7.5	12.7	12.0	9.3	8.1	50.4	100.0
**Unmet need**	14.5	23.0	19.9	11.6	7.4	5.4	18.2	100.0
Spacing	19.7	29.4	23.0	11.6	6.3	3.9	6.1	100.0
Limiting	9.2	15.5	15.9	12.5	9.8	8.1	29.0	100.0
None	9.3	19.7	15.9	11.5	8.3	6.3	28.9	100.0
Infecund	0.0	2.2	1.8	1.3	1.5	1.6	91.6	100.0
**Intention to use**								
Using already		13.9	18.6	14.2	9.9	7.3	36.1	100.0
Intends to use	25.6	29.0	13.9	7.4	4.9	3.5	15.7	100.0
Does not intend	7.2	13.1	10.6	7.0	5.4	4.5	52.2	100.0

Rapid changes occur in women's lives as their youngest child ages.

After pregnancy, women reporting amenorrhea are prominent in the first year, but that proportion declines rapidly. Afterward, contraceptive use increases, and it remains important in all intervals (women who are both amenorrheic and using a method are counted as users). Women are also divided into those who have an unmet need (and again by whether they intend to use contraception in the future or not) and those without unmet need (and whether they intend to use contraception in the future).

Total unmet need (sum of the first 2 bars below contraception for both those intending to use and not intending to use) grows substantially across the intervals. Total intention to use (first and third bars below contraception) is less than total unmet need, but it remains at a near constant level after the first year until shrinking in the final interval. Notably, the largest of the 4 subgroups pertains to women classified with unmet need who say they do not intend to use a method, which underscores the importance of watching trends for intention to use regardless of unmet need.

Infecundity in Figure 4 is not important until the final interval, which includes many of the oldest women.

Figure 4 and Figure 5 are snapshots across the intervals. In contrast, flows through time would show a great deal of movement in and out of categories for contraception and by the methods used, as well as in and out of the categories for unmet need and intention to use.

We do not show the systematic shift across the intervals from unmet need for spacing to unmet need for limiting. As noted in the Methods section, alternative definitions of unmet need have been proposed, but we are constrained by the available data to using the standard DHS definition. In the early intervals after birth, the need for spacing births dominates, but the 2 needs are about equal by the third year. Afterward, the need for limiting takes precedence. In the final interval, unmet need for spacing is nearly zero. This finding carries administrative implications for commodity requirements and budgetary adjustments.

Next, Figure 5, which focused on changes within the first year, shows rapid transitions in the mix of statuses. It separates women by the time that has elapsed since their last birth and shows the dominance of amenorrhea in the early months after birth. Its proportion shrinks through the following intervals, being largely replaced by contraceptive use. The 2 unmet need segments grow, leaving very small shares for women with no need and no intention to use and for infecundity.

Supplement 2 provides for each region a set of changes across the intervals for pregnancy, contraceptive use, unmet need crossed by intention to use, and infecundity.

### Key Subgroups Are Spread Differently Across the Intervals

Above we asked what *proportion of women* in each interval were using contraception, intending to use, or having an unmet need. Now we ask a different question: how are all contraceptive *users* distributed across the intervals; how are those women with an intention to use distributed; and how are women with an unmet need distributed (Table 2). For program purposes, it is important to know how these groups are spread according to the age of the youngest child and how they change from one survey to the next. Brief notes follow for each of the 3 groups.

#### Contraceptive Use

The type of contraceptive use changes as women move toward the intervals further from their last birth. Nonusers are concentrated among those pregnant or in the first year after birth; afterward, the decline in nonuse is quite marked, and it continues to fall off until the final group at 60+ months, where it is quite large, partly reflecting the increase in infecundity and menopause. A regular shift occurs among users of traditional methods through time; this shift is also apparent among users of short-acting methods. Balancing these shifts is the pattern for long-acting methods. Their dominance after the 5-year point reflects a dual process: as time goes by, women tend to choose a longer-acting method, and women who choose a long-acting method automatically extend their intervals.

As time goes by, women tend to choose a longer-acting method, and women who choose a long-acting method automatically extend their intervals.

#### Unmet Need

Some pregnant women are considered to have unmet need. Unmet need occurs if they did not want the current pregnancy/last birth at all (unmet need for limiting) or wanted the current pregnancy/last birth to occur at a later time (unmet need for spacing). Otherwise, they are regarded as having no unmet need.

We found that of all women with unmet need, 14.5% were currently pregnant. Another 23% fell within the first year after birth and 19.9% in the second year. These 3 groups accounted for 57.4% of all unmet need. This percentage creates a division of roughly half the unmet need being in the very early intervals for mothers with a child below age 2 and the other half among those with older children.

The need for spacing as opposed to limiting shifts systematically with longer intervals after birth. For currently pregnant women, the ratio is 19.7% for those wanting to space births against 9.2% who want to limit births. That ratio reverses sharply to 6.1% vs. 29.0% in the final interval. These shifts and differences among married women contain significant information for the mix of needed services.

The need for spacing as opposed to limiting shifts systematically with longer intervals after birth.

#### Intention to Use a Method

A woman's own declared intention to use or not to use a contraceptive method has advantages over the unmet need estimate, which is a statistical construct based on several variables. The 2 measures overlap only partly; some women classified with unmet need do not intend to use, and some women who intend to use do not have unmet need.

Over half of those who intend to use a method are in the early intervals, being either pregnant or in the first year after birth. Among those already using a method, 13.9% are in the first year, nearly 47% are within the first 3 years, and a third are in the final interval, a fairly wide spread. The distribution for those not intending to use, with half in the final interval, reflects a mix: some are not interested, some lack access to services, some are already menopausal/infecund, and some have other reasons.

**FIGURE 6:**
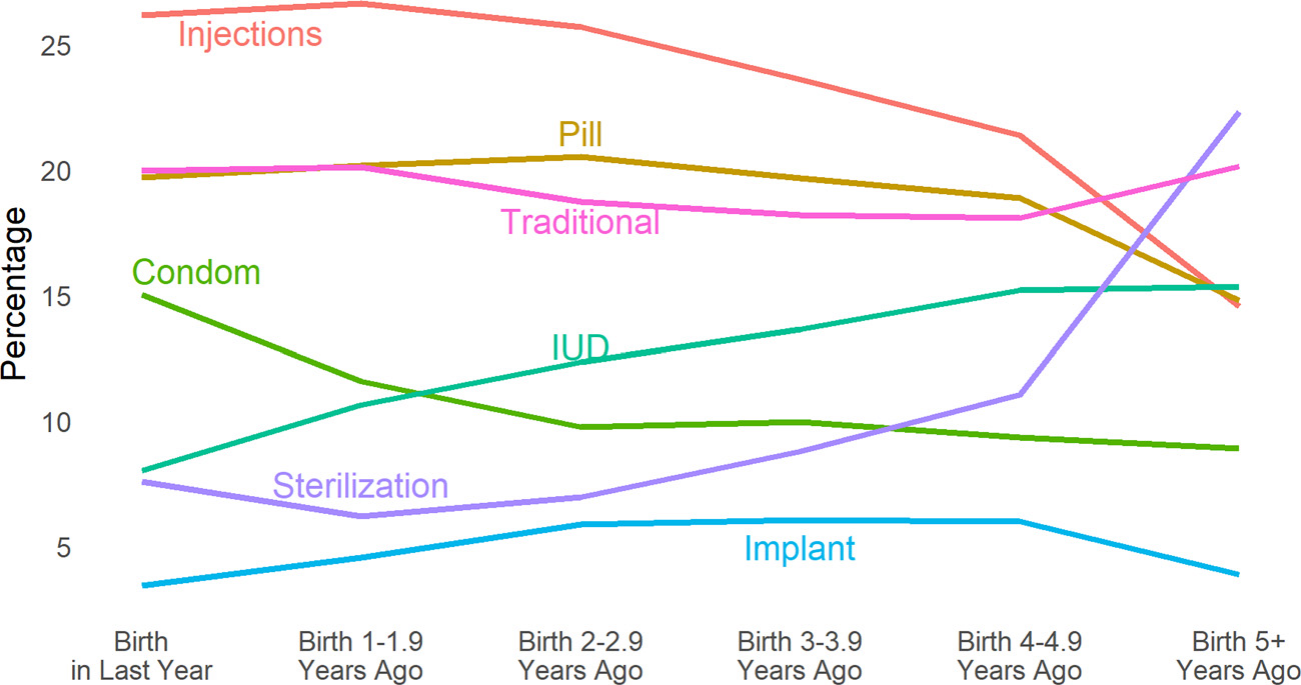
Contraceptive Method Mix by Open Birth Interval: 74 Countries, Latest Demographic and Health Surveys, Various Dates Abbreviation: IUD, intrauterine device.

Examining the contraceptive method mix in more detail is always useful because it changes in important ways as the youngest child ages. As Figure 6 shows, the injectable, pill, and traditional methods play the strongest roles through most of the intervals, but injectables clearly decline in the later intervals. Condom use also starts strong but declines quickly. Meanwhile, use of the intrauterine device (IUD) increases steadily, as does sterilization (both sexes), which is the most common method by the final interval. All methods add to 100% in each interval.

Thus, women tend to adjust their choice of methods as they and their youngest child age. Although the specific pattern may vary across regions and by personal characteristics, trend information on the open interval can provide useful information for each country regarding changing method preferences. Finally, the pattern pertains to current users, not annual adoptions. The percentage using sterilization reflects an accumulation of past adoptions; that is true to a lesser extent for the IUD and implant. On the other hand, current use of all spacing methods reflects adoptions in the recent past.

### Demographic Groups Show Systematic Patterns Across the Intervals

Systematic changes occur across the open intervals according to personal characteristics because they correlate broadly with reproductive health needs (Supplement 3). By age, the older women are, the longer the time that has elapsed since the latest birth. Women aged 15–19 cluster as either pregnant or in the first year, while women aged 45–49 are nearly absent in all intervals except the last one. By number of living children, the pattern mirrors that for age. The shift in the interval since the last birth is perfectly regular: more recent for women with 1 child to more distant for those with 4 or more. By residence, the differences are not large, but they run in the expected direction: rural women with their higher fertility rates fall within the more recent intervals, with more recent births. Finally, by wealth quintiles, the contrasts are entirely regular: the poorer the women, the more likely they are pregnant or have an infant in arms. Among the wealthiest women, the youngest child is older than in any other group. It should be noted that the measure of wealth is defined by the household in which the woman lives.

Systematic changes occur across the open intervals according to personal characteristics because they correlate broadly with reproductive health needs.

### Changes Over Time for Percentage Distributions Can Mask Changes in Numbers of Women

Changes in the percentages of women within each of the open interval categories do not necessarily translate into similar changes for absolute numbers of women. Trends for numbers arise from both population growth and the trends in the percentages. In general, we expect that the growing number of women in the population will offset declines in the percentages of pregnant women and often override them. Consequently, the absolute numbers may actually increase, even while the percentages decline for women who are either pregnant or in the first year after birth.

**FIGURE 7:**
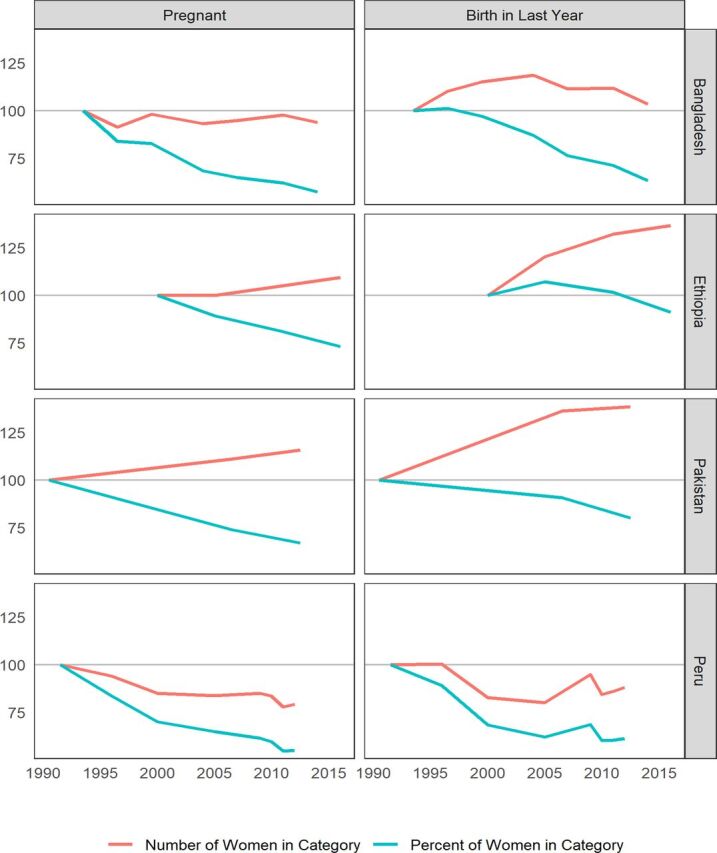
Changes in the Percentage of Reproductive Age Women Who Are Pregnant or in the First Year After Birth, in Bangladesh, Ethiopia, Pakistan, and Peru

The 4 countries in Figure 7 were chosen to illustrate the various patterns that can arise. The earliest survey serves as an index of 100. The left panel, for pregnant women, contrasts the relative trends for percentages versus numbers, and the right panel, for women in the first year after birth, shows similar contrasts.

- Bangladesh's long-term increase in contraceptive use has markedly reduced the percentage of women who are pregnant or in their first year after birth. Early and continuing fertility declines have attenuated growth in the number of childbearing women, so the numbers pregnant have stayed roughly stable.
- The picture is far different in Ethiopia and Pakistan. These countries have experienced 30% to at least 35% declines in the percentage of women who are pregnant, with lesser declines for women in the first year after birth. However the numbers have increased: 10% to 19% for pregnant women and 35% or more for those within a year after birth.
- Peru presents a different case. Both the percentages and the numbers have declined, by roughly 40% for those pregnant or those within a year after birth, accompanied by significant declines in numbers. That pattern emerges from an early and sharp increase in contraceptive use between 1991 and 2000, with a corresponding decline in the total fertility rate that modified the age structure toward relatively fewer women of childbearing age by 2012 (latest survey). Total contraceptive use rose from 40% in 1986 to 70% and higher after 2000, while the total fertility rate fell from 4.1 to about 2.5 in the same years.

These cases show that managers must track numbers, not just percentages, as they change through time. Numbers may grow within all intervals, but not proportionately. Instead, as fertility and pregnancy rates decline they may grow much less in the early intervals.

### Relationships Are Close Between the Open Interval and Other Fertility Measures

The open interval offers its own advantages compared with the familiar ones of the total fertility rate (TFR), the general fertility rate (GFR), and the age-specific fertility rates (ASFR). These rates commonly pertain to births over the previous 3 to 5 years, as in the DHS reports, but the open interval as a survey snapshot can reflect the current state due to both recent births and the behavior over many past years that produced the older children.

Further, changes in the open interval and in the other rates behave somewhat differently as annual births occur. More births elevate the GFR and TFR, but they affect the open interval distribution mainly only at the start, where the births are located. A burst in the fertility rate tends to increase women in the first interval, but other parts of the distribution can change if births start coming more than usually from women located in the later intervals. In addition, if more women than usual in the final interval age out, that can modify the distribution. In general, however, the shape of the distribution is relatively stable.

Closed intervals, as valuable as they are, omit much reproductive behavior. Many women are actively avoiding pregnancy and birth and will never have another. Most women who have gone 5 years without a birth tend not to have another; 84% of closed intervals are less than 5 years long (mean across 298 DHS surveys in the STATcompiler, accessed July 22, 2018).

A very close correlation exists between the open interval and the usual fertility rates. For the 74 countries in the current study, the correlations are 0.93 to 0.97 between the GFR or TFR and such open interval measures as the percentage of women who are within the first year after birth, or equally, the percentages within 2 years, or 3, 4, or 5 years after birth (Table 3). Those correlations are all positive except the last one: the more women in the earlier intervals, the higher the fertility rate. But for intervals of 5 years or more, the correlation (0.93) reverses direction: the more women going without a birth for a long time, the lower the fertility rate.

**TABLE 3.** Correlation Coefficients Between Open Interval and the General Fertility Rate and the Total Fertility Rate: 74 Countries, Latest Demographic and Health Surveys, Various Dates^a,b^

Length of Open Intervals (months)	General Fertility Rate	Total Fertility Rate
<12	0.96	0.97
<24	0.96	0.97
<36	0.96	0.97
<48	0.95	0.96
<60	0.93	0.94
60+	(0.93)	(0.94)

a *r* values, when squared give *R*^2^ values.

b Interval data are from the latest surveys in the 74 countries; fertility data are as issued by the United Nations as of approximately 2017.

Given this close association between the open interval and the TFR or GFR, the single question on when the last birth occurred provides an added picture of fertility behavior, one that has its own advantages and is free of the multiple questions needed to calculate the TFR and GFR.

The single question on when the last birth occurred can provide an added picture of fertility.

Finally, the average level of the TFR is clearly associated with the open interval. Countries averaging a TFR below 3 have well over half (56%) of married women with the youngest child over age 5. At the other extreme, countries averaging TFRs over 5 have only a fifth (20%) in that bracket. In between the parallels are exact (Table 4).

**TABLE 4.** Distribution of Women of Reproductive Age by the Length of the Open Birth Interval, According to the Total Fertility Rate: 74 Countries, Latest Demographic and Health Surveys, Various Dates^a^

TFR	Open Intervals (Months)						Total
	Pregnant/1–11	12–23	24–35	36–47	48–59	60+	
<3	14.2	9.4	7.9	6.7	5.8	55.9	100.0
3–3.9	23.4	13.9	10.4	7.9	6.3	38.1	100.0
4–4.9	30.6	17.2	11.6	8.2	5.9	26.7	100.0
>5	37.8	20.0	11.0	6.5	4.5	20.3	100.0

Abbreviation: TFR, total fertility rate.

a Interval data and TFRs are both from the latest surveys in 74 countries.

### A Simple Model Captures the Open Interval Distribution

The shape of the open interval distribution is remarkably similar across countries. Because the pattern is nearly universal, a model with just 2 parameters captures the level and the sharpness of the decline in numbers of women as the intervals since the last birth become longer. These 2 parameters, labeled *a* and *b* in a power equation, are discussed in Supplement 4 and the values of the parameters are provided for all surveys in the 74 countries. Supplement 4 also shows how the shape of the open interval distribution can change over time; illustrated by Rwanda, which has had a strong anti-natalist national policy with vigorous implementation. On average, between 1992 and 2005, 44% of married women were either pregnant or in the first year after birth; that fell to a remarkable 30% in the 2010 and 2014 surveys.

### Women's Empowerment Is Related to the Age of the Youngest Child

Upon release from child care, women have more options for activities outside of the home. They can explore or widen roles other than pregnancy—different ones from the homebound duties tied to motherhood. Some of the new options are economic in nature, and one way to examine these changes is by the degree of women's participation in the labor force. We used data from the International Labor Organization on women's participation in the labor force to compare the rate of their participation to the proportion of women whose youngest child is over age 5. The results are based on a fixed effects analysis, which controls for spurious cross-country correlations. For example, compared with other regions, Asian countries tend to have both longer intervals since the last birth, suggesting greater female freedom, and lower rates of labor force participation, running counter to the relative levels in other regions. But within individual countries, the association is generally positive between the open interval and female participation in the labor force.^11^ When the changes over time within individual countries are averaged, a positive correlation results, for a ratio of 10:4; that is, for a 10 percentage point increase of women whose youngest child is over age 5, there is a 4 percentage point increase of women in the labor force. Against a global average of 39% of women in the labor force, that increment is notable.

**FIGURE 8:**
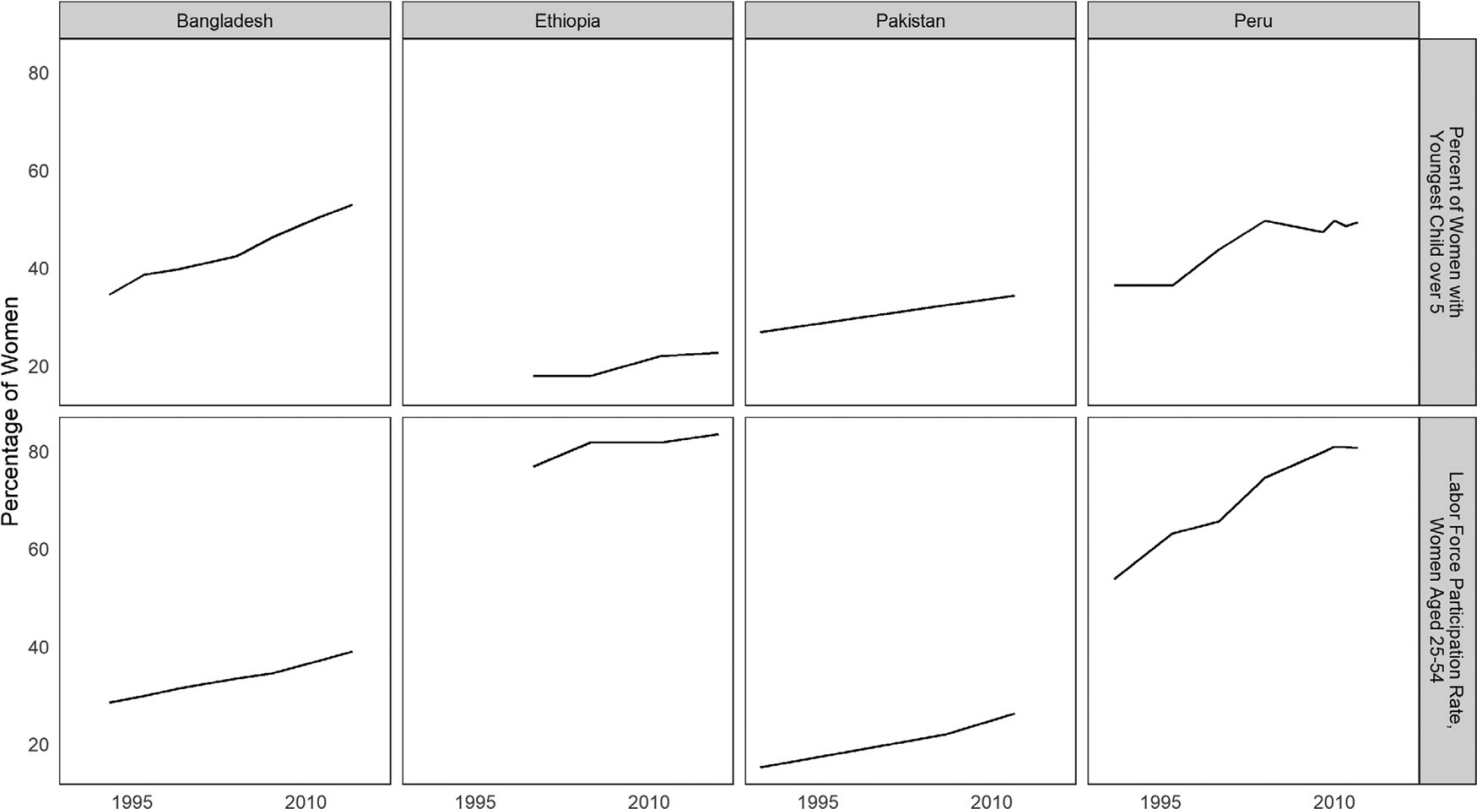
Comparison of Changes in the Percentage of Women Whose Youngest Child Is Over Age 5 and Changes in the Percentage of Women in the Labor Force, in Bangladesh, Ethiopia, Pakistan, and Peru

The parallel trends of female participation in the labor force and the age of the youngest child are shown in Figure 8, in which 4 countries were chosen to illustrate different country situations from 3 regions. The top panel, drawn from the DHS, is matched by date to labor force information in the lower panel. A faster pace in both respects is evident for Bangladesh in comparison with Pakistan, but Peru outpaces both, especially in the high and rising growth of women in the labor force. Ethiopia represents a sub-Saharan African country with many women in the labor force, despite having the lowest percentage of women whose youngest child is over age 5. These are some of the country contrasts that lie behind the average 10:4 ratio.

In general, the balance of home roles and outside roles changes as the youngest child ages. Women can engage more in formal employment and small business ventures, with more personal income and enhanced power within the family. They can be out in the greater world, with enhanced freedom of movement, seeking job training and further education and making wider social connections. Such changes tend to enhance both gender equity and female empowerment.

As the youngest child ages, women gain more opportunities and a greater role in economic development.

The development process can also be hastened by greater female equity in these respects. The World Bank notes that^12^:

*… gender equality is a core development objective in its own right. But greater gender equality is also smart economics, enhancing productivity and improving other development outcomes. It urges closing of gender gaps in access to economic opportunities, earnings, and productivity.*

Further, the World Bank asks for a reduction of gaps in human capital, specifically those that address female mortality and education. It notes that^12^:

*In nearly every country today, women face barriers to fully participate in the work force and earn as much as men. Because of this, women account for only 38 percent of their country's human capital wealth, defined as the value of the future earnings of their adult citizens—versus 62 percent for men. In low income and lower-middle income countries, women account for just a third or less of human capital wealth.*

**FIGURE 9:**
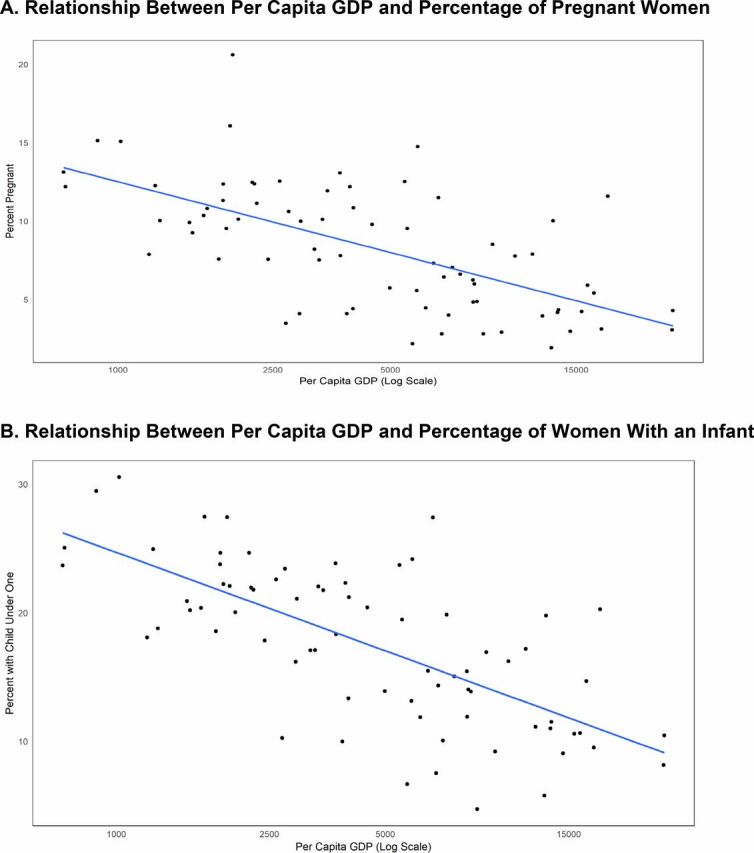
Relationship Between Gross Domestic Product (GDP) per Capita and Women's Childbearing

The development process and the roles of women are intertwined in many respects, including the extent of their involvement in childbearing. To explore this relationship, we examined the association between GDP (gross domestic product) per capita and the percentages of women either pregnant or with an infant (up to 1 year old). The relationships across the 74 counties in our analysis appear in Figure 9A and Figure 9B, first for lower pregnancy rates and second for presence of an infant. The figure shows the negative association between the 2 with GDP per capita income (*R*^2^ values 0.40 and 0.48, respectively). All 3 are in turn related to the prevailing fertility rates (not shown) since lower fertility is associated with high GDP per capita and also with the 2 measures of child care. Overall, the associations in the figure are consistent with a link between faster national development and the percentage of women free from early child care.

These issues are intertwined. Reductions in child care encourage labor force participation, which in turn often leads women to postpone or avoid a next birth. Women can bring advantages to the economy and can hasten the development process, which then tends to enlarge job opportunities for more women to enter selected occupations. The results of these processes can serve to advance women's empowerment.

### Program Designs Can Benefit From Open Interval Information

Each part of the open interval distribution tells a story that programs can benefit from. Changes in the proportions of women who are pregnant or have recently given birth signal necessary modifications in the allocation of the services needed. These pertain especially to maternity care, postpartum services, and early contraceptive offerings. But equally, attention to the numbers in the intermediate and later intervals can clarify the likely need, or market, for the changing mix of contraception shown above between short-acting and long-acting methods.

Each part of the open interval distribution tells a story that programs can benefit from.

Absolute numbers within the various intervals are important for planning, as are rapid changes in method mix from one survey to the next in particular intervals, as they have been in sub-Saharan Africa toward the implant and injectable.^13^ Close monitoring for these methods, along with the numbers involved, enables modifications to supply lines, personnel allocations, clinic operations, and budgets by type of expenditure.

Asking about the age of the youngest child is useful for service staff who provide family planning. Counselors and fieldworkers may already ask about the age of the woman's youngest child, but that information is not captured in the usual service statistics systems that are fed upward for management purposes. It is available only in surveys at national and lower levels. Where not already systematically done in client contacts, establishing the youngest child's age provides a springboard to ask about intentions for spacing or limiting in counseling about contraceptive methods, and it affords an opportunity to enquire about key health services for the child such as immunization.

In rural and peri-urban settlements, where many women lack easy access to services, workers should pay particular attention to women with very young children because they are the most likely to have early, unplanned pregnancies. All outreach activities should recognize that a woman's need for, readiness for, and interest in contraceptive use is tied closely to the age of her youngest child.

#### Postpartum Avenues

The “extended postpartum period,” the first year after birth, has been of particular interest since the 1960s.^14^^,^^15^ Most women after a birth do not want another one quickly,^16^ and at delivery most are in immediate contact with the needed services, as well as later at the 6-week checkups. While many women will avoid another conception during much of the first year owing to amenorrhea, delays often occur before adoption of contraception, and the most fecund “early conceivers” will often have unplanned pregnancies. Some overlap of contraceptive protection with amenorrhea can occur, but given the downside risk of an unplanned pregnancy, the better strategy usually lies with adoption of a method relatively soon. Further, access to the method before leaving the hospital is important for those who will not be seen again. Programs must work in the large and cannot be fine-tuned to the return of menses for the individual woman. The rationales for the programs have also included the health benefits of adequate birth spacing, and linkages at or soon after birth to parts of the health system for women, both for themselves and for preventive services for their child, notably immunization.

Meanwhile, attention continues to be focused on “best practices” for the implementation of these programs, as in a review by Cleland et al.^17^ They examined the effects of 35 interventions by time and type (antenatal, postnatal, both, and integration with other services), finding generally positive impacts of the interventions. The evidence was regarded as incomplete but still useful for guidance to advance postpartum programs in different contexts.

In general, program implementation can only gain by knowledge of where women are within the open interval distribution and how the distribution has changed since the previous survey. Both proportions and numbers need attention, especially since the absolute numbers of women in the population diminish sharply in the later intervals due to aging, and because progressively more women are infecund or already using contraception. The largest absolute numbers of women are in the early and intermediate intervals.

Program implementation benefits by knowledge of where women are within the open interval distribution and how the distribution has changed over time.

#### Beyond Postpartum

After the postpartum period, attention must go to the middle and later intervals. Over 60% of unmet need for family planning and over 40% of intention to use contraception fall beyond 1 year postpartum, as shown in Table 2. Moreover, over time fewer and fewer women may have recent deliveries, shifting more women into the later intervals (Figure 2). Effective family planning programming requires improved access to all methods and over all intervals, as well as through a maximum variety of avenues. Broad-based programs should embrace such modalities as mobile outreach, social franchising, community-based delivery of contraception, postabortion care, and behavior change communication in communities to enhance information and demand.

The survey data show that substantial percentages of women want to postpone a next pregnancy for 2 years or longer, so even for those who wish to space rather than limit, the reversible long-acting methods have a role to play, especially given their low failure and discontinuation rates. Van Lith et al.^18^ found that even in sub-Saharan Africa (18 countries) demand for limiting exists among 14% of women, and for spacing among 25%, and that among all married women, the 2 are nearly equal. Women wishing to limit are an unappreciated subgroup for whom longer-acting methods are being neglected. Jacobstein^19^ found that the implant now ranks first or second among all methods in 10 countries, reflecting sizeable price reductions, increased commodity supply, broader World Health Organization eligibility guidelines, and improved service delivery practices. These illustrate the potential of service improvements in combination with long-acting methods that can assist women across all of the open intervals.

Ministries of health have additional channels to broaden access to contraceptive assistance. While family planning efforts have focused on integration with immunization services for children, little attention has been paid to linkages with curative health services for them. Unlike immunization, curative treatments continue throughout childhood at a variety of clinics for such ailments as diarrhea, pneumonia, injuries, and infections. The mothers rather than the fathers usually accompany children needing attention, and finding creative ways to link family planning to these services deserves fresh thinking, especially given current emphases on primary health care. For the mothers, the growth of cancer screenings is an additional service of interest.

## DISCUSSION AND CONCLUSIONS

The simple question “How long has it been since your last birth?” differentiates women in a fundamental manner, by the age of their youngest child. Easily available over time in national surveys, the open birth interval shows movement through the stages of reproductive behavior, it informs fertility analyses, and it offers guidelines for national action programs. In addition, it relates to women's movement into the labor force and to policies for women's empowerment.

The open birth interval shows movement through reproductive stages, informs fertility analyses, and offers guidelines for national action programs.

For the first time, a large body of empirical information on the open birth interval has been assembled and analyzed. This study shows that the distribution has a characteristic shape, with a substantial proportion of women near a birth or expecting one soon, then declining proportions through 15 years and beyond. While this characteristic shape is present everywhere, the relative proportions between the first year and the later years varies a great deal, from countries with very low fertility and therefore few women either pregnant or in the first year after birth, to countries with high fertility rates and therefore many women who are pregnant or in the first year after birth. The simple model presented in Supplement 4 captures these changes and allows for estimates across countries and over time.

The age of a woman's youngest child carries important implications for her freedom of action, and it varies greatly across regions. In sub-Saharan Africa at one extreme, and in the European/West Asia countries at the other, women are preoccupied to greater and lesser degrees with pregnancy and childrearing. Correspondingly, they vary in the ages at which they are able to pursue other roles. Much of that is captured in the distribution of delays since the latest birth, as women's circumstances fundamentally change as their youngest child moves from infancy to childhood to school and finally departs from the home. In between, the needed health and social services evolve in character.

For public programs devoted to reproductive health, the distribution of women along the axis of their youngest child, and the absolute numbers within each of the early intervals, are basic for planning. Information by year within the open interval is unique; it gives insights not present in averages and overall estimates.

Policy makers should examine open birth interval data in making economic development policy. They should recognize that providing voluntary family planning services not only benefits individual women but also advances overall economic development. Considerations of social policy and equity for women can only benefit from information on the proportions of women preoccupied with childbearing and the extent to which women can enter the labor force. Those estimates and the changes from one survey to the next are relevant to advocacy efforts to reduce barriers to equal earnings and opportunities for the advancement of girls and women.

We recommend that national planners for reproductive health programs and social policy examine each new survey for the open birth interval distribution and its correlates, in light of changes since the previous surveys. This information will augment other bodies of information currently in use to strengthen both the planning and the implementation of national programs.

## Supplementary Material

19-00056-Ross-Supplement2.xlsx

19-00056-Ross-Supplement1.xlsx

19-00056-Ross-Supplement4.pdf

19-00056-Ross-Supplement3.pdf

## Notes

**Peer Reviewed**

**Funding**: This article is funded by the Track20 Project of the Avenir Health organization, which is supported by the Bill & Melinda Gates Foundation.
