# Supplementary material for: The Open Birth Interval: A Resource for Reproductive Health Programs and Women's Empowerment
Source: Glob Health Sci Pract. 2019 Sep 23;7(3):355–70. doi: 10.9745/GHSP-D-19-00056 (PMC6816813; doi:10.9745/GHSP-D-19-00056)
Supplement: 19-00056-Ross-Supplement4.pdf [file 19-00056-Ross-Supplement4.pdf]

Ross J, Bietsch K. The open birth interval: a resource for reproductive health programs and women's empowerment. Glob Health Sci Pract. 2019;7(3). https:/doi.org/10.9745/GHSP-D-19-00056

SUPPLEMENT 4. Curve-Fitting of the Open Interval Distribution

In early tabulations we noticed that the shape of the open interval distribution was markedly similar across countries. The married women were clustered close to the shorter intervals since their last birth, with regularly descending percentages toward the longer intervals. We therefore questioned whether a simple fitted curve with only a few parameters might be possible.

Using all 15 intervals (from the first year to 15 years) we generated curves like those shown in the following figure for Rwanda at various dates.

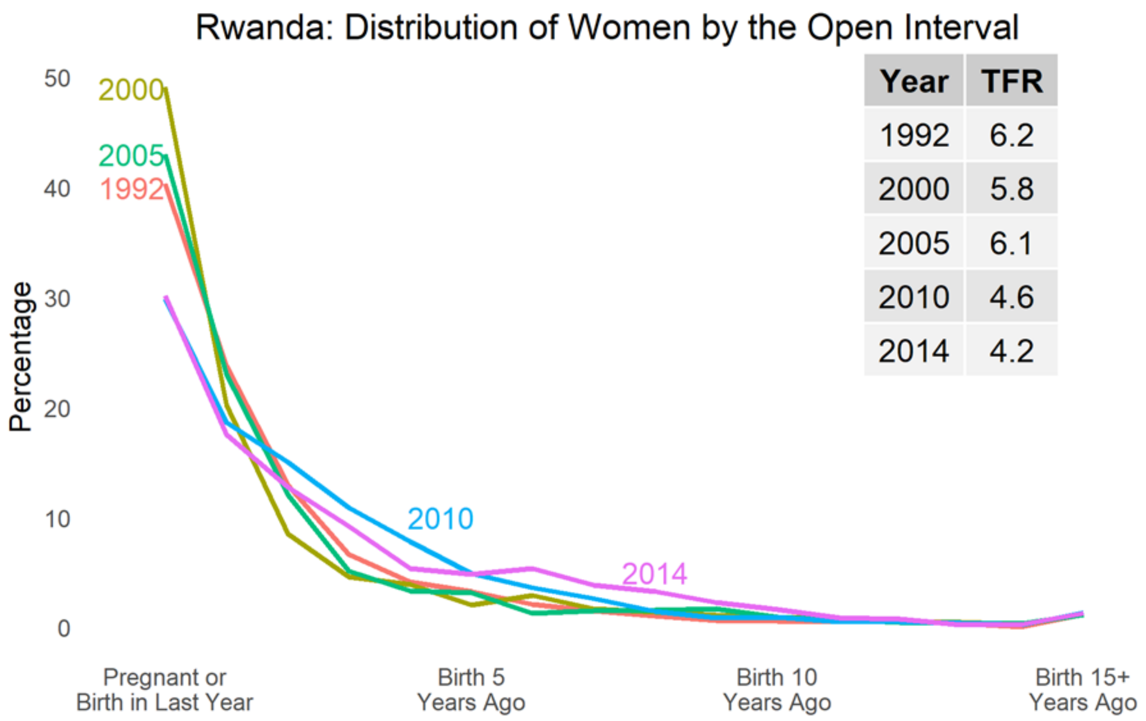

(Note that the 2010 and 2014 lines start at identical points, for 30%).

The dominant pattern in all other countries is the same. Women are concentrated to the left. Then the curve descends, at varying slopes depending upon the prevailing fertility level, as shown for the TFR values in the Rwanda figure. The descent is due partly to fewer women at the upper ages, where the open interval is longer.

Because this pattern is so universal, we fitted various equations to the typical shape, and found a "power" equation to provide a very close fit. It is of the form Y = "a" times (X taken to the "b" power) or $Y=a(x^b)$. It is a negative exponential curve as in the Figure, in which the curve falls off faster with a larger "b" value. The curve as a whole is placed higher or lower according to the "a" value.

A close relation exists between the open interval distribution and the usual fertility measures of the GFR and the TFR. The following Matrix ("r" values, which give R2 values if squared) shows the correlations to be above 0.90 across the 74 countries with the latest surveys.

Matrix for Correlations Between Fertility and Open Interval

	"a" Value	*"b" Value*	*GFR*	*TFR*
"a" Value	1.00			
"b" Value	0.98	1.00		
GFR	0.92	0.94	1.00	
TFR	0.93	0.95	0.99	1.00

(The matrix gives "r" values, which give R2 values when squared)

Trends: Over time the average values have moved toward lower fertility levels. The following table (bottom row) shows the percentage decline of 16% to 17% in the GFR and TFR in the 56 countries with multiple surveys, over an average period of 17 years. Changes in the "a" and "b" values, at 13% and 11%, have been basically similar. The "a" value estimates the proportion of women in the first interval, i.e. the level at the start of the curve. The decline was from about 42% to about 37% of women in that interval (first column). The change in the "b" value, indicates a softening in the slope of the curve, with fewer women at the start and more toward the longest intervals. (The R2 values hardly changed at all, most being well above 0.900.)

Summary of Changes in the "a" and "b" Values and in GFR and TFR

	"a" Value	"b" Value	R2	GFR	TFR
Earliest Surveys	0.416	(1.287)	0.949	165	4.8
Latest Surveys	0.369	(1.147)	0.944	140	4.0
Change	(0.047)	0.140	(0.004)	(25)	(0.8)
% Change	(12.6)	10.6	(0.2)	(15.7)	(16.8)

The search for summary measures of the full 15-year distribution of intervals can use the "a" and "b" values, along with such others as the percentage of intervals less than one year, two years, etc. Succinct summary measures can be employed to examine such determinants as age, family size, and residence, along with contraceptive use, intention to use, and unmet need. Separately, a study of the rapidity of change within individual countries is of interest.

The two summary measures, "a" and "b", appear in the following table; it pertains to the 74 countries used above and includes the latest surveys for those countries, plus 56 more for the earliest surveys in countries having multiple surveys, making 130 total. The table gives the R2 correlations between the "a" and "b" values, and also includes the general fertility rate (GFR) and the total fertility rate (TFR). Anyone can generate the full estimated curve along the X-axis of 15 years by plugging the "a" and "b" values into the equation for any country.

Table for Curve Fitting: "a" and "b" Values, Their R2 Correlation, GFR and TFR

This table lists 130 surveys: 74 for the latest surveys in 74 countries, and 56 more for the earliest surveys in countries with multiple surveys.

	"a" Value	"b" Value	R2	GFR	TFR		"a" Value	"b" Value	R2	GFR	TFR
Afghanistan 2015	0.440	-1.426	0.956	175	5.3	Kyrgyz Republic 1997	0.229	-0.82	0.917	118	3.4
Albania 2008-09	0.065	-0.132	0.390	46	1.6	Kyrgyz Republic 2012	0.257	-0.968	0.985	125	3.6
Angola 2015-16	0.603	-1.678	0.970	216	6.2	Lesotho 2004	0.318	-1.072	0.970	121	3.5
Armenia 2000	0.079	-0.251	0.672	56	1.7	Lesotho 2014	0.312	-1.05	0.929	118	3.3
Armenia 2015-16	0.121	-0.538	0.968	64	1.7	Liberia 2007	0.412	-1.303	0.958	181	5.2
Azerbaijan 2006	0.089	-0.328	0.600	66	2.0	Liberia 2013	0.418	-1.282	0.935	168	4.7
Bangladesh 1999-00	0.333	-1.076	0.918	130	3.3	Madagascar 1992	0.455	-1.483	0.979	212	6.1
Bangladesh 2014	0.165	-0.63	0.943	90	2.3	Madagascar 2008-09	0.396	-1.255	0.958	168	4.8
Benin 1996	0.548	-1.579	0.974	202	6.0	Malawi 1992	0.470	-1.514	0.956	223	6.7
Benin 2011-12	0.508	-1.475	0.956	175	4.9	Malawi 2015-16	0.515	-1.445	0.914	158	4.4
Bolivia 1994	0.397	-1.255	0.970	163	4.8	Maldives 2009	0.228	-0.827	0.962	82	2.5
Bolivia 2008	0.281	-0.963	0.913	121	3.5	Mali 1995-96	0.529	-1.585	0.979	234	6.7
Brazil 1991	0.268	-0.932	0.924	122	3.4	Mali 2012-13	0.559	-1.59	0.967	214	6.1
Brazil 1996	0.147	-0.538	0.931	89	2.5	Moldova 2005	0.078	-0.294	0.747	55	1.7
Burkina Faso 1993	0.691	-1.804	0.946	221	6.5	Morocco 1992	0.412	-1.294	0.981	127	4.0
Burkina Faso 2010	0.581	-1.616	0.961	206	6.0	Morocco 2003-04	0.262	-0.912	0.941	81	2.5
Burundi 2010	0.732	-1.942	0.971	203	6.4	Mozambique 1997	0.440	-1.407	0.991	183	5.2
Burundi 2016-17	0.748	-1.846	0.923	180	5.5	Mozambique 2011	0.458	-1.464	0.975	206	5.9
Cambodia 2000	0.376	-1.178	0.868	119	3.8	Myanmar 2015-16	0.198	-0.723	0.932	77	2.3
Cambodia 2014	0.215	-0.779	0.953	98	2.7	Namibia 1992	0.410	-1.325	0.957	176	5.4
Cameroon 1991	0.437	-1.417	0.972	208	5.8	Namibia 2013	0.305	-1.039	0.948	125	3.6

Cameroon 2011	0.426	-1.385	0.977	180	5.1	Nepal 1996	0.320	-1.097	0.976	167	4.6
Central African Rep. 1994-95	0.428	-1.388	0.966	183	5.1	Nepal 2016	0.146	-0.573	0.970	88	2.3
Chad 1996-97	0.562	-1.679	0.955	229	6.4	Nicaragua 1998	0.284	-0.976	0.973	132	3.6
Chad 2014-15	0.597	-1.697	0.954	230	6.4	Nicaragua 2001	0.258	-0.91	0.906	117	3.2
Colombia 1990	0.245	-0.875	0.956	105	2.8	Niger 1992	0.566	-1.687	0.978	250	7.0
Colombia 2015	0.129	-0.495	0.947	70	2.0	Niger 2012	0.699	-1.881	0.967	269	7.6
Comoros 1996	0.414	-1.349	0.937	148	4.6	Nigeria 1990	0.561	-1.615	0.934	207	6.0
Comoros 2012	0.392	-1.283	0.981	142	4.3	Nigeria 2013	0.473	-1.473	0.969	190	5.5
Congo 2005	0.419	-1.345	0.986	168	4.8	Pakistan 1990-91	0.441	-1.387	0.940	164	4.9
Congo 2011-12	0.471	-1.453	0.988	182	5.1	Pakistan 2012-13	0.301	-1.05	0.979	131	3.8
Congo Democratic Rep. 2007	0.459	-1.477	0.975	210	6.3	Paraguay 1990	0.395	-1.255	0.943	160	4.7
Congo Democratic Rep. 2013-14	0.535	-1.641	0.982	225	6.6	Peru 1991-92	0.301	-1.028	0.969	121	3.5
Cote d'Ivoire 1994	0.571	-1.595	0.949	187	5.3	Peru 2012	0.198	-0.746	0.911	86	2.6
Cote d'Ivoire 2011-12	0.469	-1.423	0.973	174	5.0	Philippines 1993	0.302	-1.041	0.985	138	4.1
Dominican Rep. 1991	0.247	-0.905	0.981	125	3.3	Philippines 2013	0.225	-0.824	0.940	101	3.0
Dominican Rep. 2013	0.172	-0.696	0.977	89	2.5	Rwanda 1992	0.888	-2.06	0.919	197	6.2
Egypt 1992	0.353	-1.136	0.959	136	3.9	Rwanda 2014-15	0.646	-1.629	0.832	142	4.2
Egypt 2014	0.271	-0.971	0.985	127	3.5	Sao Tome and Principe 2008-09	0.457	-1.392	0.952	164	4.9
Ethiopia 2000	0.588	-1.655	0.940	183	5.5	Senegal 1992-93	0.504	-1.536	0.974	202	6.0
Ethiopia 2016	0.544	-1.506	0.925	156	4.6	Senegal 2016	0.602	-1.607	0.934	156	4.7
Gabon 2000	0.300	-1.044	0.946	153	4.2	Sierra Leone 2008	0.429	-1.323	0.942	180	5.1
Gabon 2012	0.359	-1.225	0.947	144	4.1	Sierra Leone 2013	0.489	-1.428	0.938	169	4.9
Gambia 2013	0.561	-1.635	0.974	185	5.6	South Africa 1998	0.195	-0.735	0.894	100	2.9

Ghana 1993	0.498	-1.465	0.963	178	5.2	Swaziland 2006-07	0.307	-1.055	0.946	137	3.8
Ghana 2014	0.386	-1.242	0.977	143	4.2	Tajikistan 2012	0.249	-0.917	0.959	134	3.8
Guatemala 1995	0.317	-1.114	0.987	177	5.1	Tanzania 1991-92	0.480	-1.498	0.972	212	6.2
Guatemala 2014-15	0.254	-0.904	0.981	112	3.1	Tanzania 2015-16	0.482	-1.437	0.962	178	5.2
Guinea 1999	0.490	-1.448	0.963	195	5.5	Timor-Leste 2009-10	0.575	-1.579	0.923	175	5.7
Guinea 2012	0.462	-1.415	0.937	176	5.1	Timor-Leste 2016	0.384	-1.197	0.913	136	4.2
Guyana 2009	0.152	-0.602	0.914	94	2.8	Togo 1998	0.520	-1.513	0.965	175	5.2
Haiti 1994-95	0.406	-1.324	0.984	156	4.8	Togo 2013-14	0.479	-1.425	0.965	163	4.8
Haiti 2012	0.326	-1.086	0.943	117	3.5	Turkey 1993	0.177	-0.668	0.939	90	2.5
Honduras 2005-06	0.290	-0.991	0.923	117	3.3	Turkey 2003	0.160	-0.646	0.956	79	2.2
Honduras 2011-12	0.235	-0.844	0.974	107	2.9	Uganda 1995	0.592	-1.773	0.966	248	6.9
India 1992-93	0.230	-0.861	0.967	130	3.4	Uganda 2016	0.559	-1.568	0.966	189	5.4
India 2015-16	0.125	-0.52	0.968	81	2.2	Ukraine 2007	0.066	-0.254	0.798	39	1.2
Indonesia 1991	0.256	-0.906	0.929	108	3.0	Uzbekistan 1996	0.259	-0.9	0.959	123	3.3
Indonesia 2012	0.185	-0.698	0.914	88	2.6	Vietnam 1997	0.184	-0.645	0.876	80	2.3
Jordan 1990	0.476	-1.453	0.980	169	5.6	Vietnam 2002	0.106	-0.341	0.753	60	1.9
Jordan 2012	0.326	-1.08	0.967	112	3.5	Yemen 2013	0.454	-1.373	0.959	146	4.4
Kazakhstan 1995	0.144	-0.551	0.839	83	2.5	Zambia 1992	0.495	-1.557	0.971	219	6.5
Kazakhstan 1999	0.105	-0.351	0.819	67	2.0	Zambia 2013-14	0.581	-1.597	0.954	184	5.3
Kenya 1993	0.423	-1.305	0.962	182	5.4	Zimbabwe 1994	0.463	-1.373	0.951	148	4.3
Kenya 2014	0.400	-1.236	0.929	141	3.9	Zimbabwe 2015	0.470	-1.379	0.931	144	4.0
