# Supplementary material for: The Open Birth Interval: A Resource for Reproductive Health Programs and Women's Empowerment
Source: Glob Health Sci Pract. 2019 Sep 23;7(3):355–70. doi: 10.9745/GHSP-D-19-00056 (PMC6816813; doi:10.9745/GHSP-D-19-00056)
Supplement: 19-00056-Ross-Supplement3.pdf [file 19-00056-Ross-Supplement3.pdf]

Ross J, Bietsch K. The open birth interval: a resource for reproductive health programs and women's empowerment. Glob Health Sci Pract. 2019;7(3). https:/doi.org/10.9745/GHSP-D-19-00056

SUPPLEMENT 3. Open Interval Distributions by Personal Characteristics

Based on mean values for the latest surveys in the 74 countries. For brevity of display the intervals from 60+ months onward are collapsed.

Profiles of Married Women by Open Intervals and Personal Characteristics

	Open Intervals (Months)						
	**Pregnant to 11 mo.**	**12 - 23**	**24 - 35**	**36 - 47**	**48 - 59**	**60+**	**Sum**
ALL WOMEN	25.6	14.8	10.1	7.2	5.6	36.7	100.0
AGE							
15-19	63.7	24.9	8.3	2.3	0.6	0.3	100.0
20-24	46.5	24.9	13.7	7.5	3.7	3.6	100.0
25-29	35.8	20.6	13.9	9.3	6.5	14.1	100.0
30-34	27.2	16.0	12.2	9.0	7.2	28.4	100.0
35-39	18.4	11.8	9.5	8.0	6.9	45.5	100.0
40-44	8.4	6.5	6.6	6.5	6.3	65.6	100.0
45-49	2.1	2.0	2.5	3.2	3.6	86.7	100.0
No. of LIVING CHILDREN							
1	36.3	17.5	10.7	6.8	4.5	24.2	100.0
2	28.2	15.6	10.5	7.0	5.1	33.6	100.0
3	24.5	14.4	9.6	7.0	5.7	38.7	100.0
4+	20.2	13.1	9.5	7.3	6.2	43.7	100.0
RESIDENCE							
Urban	23.2	13.5	9.9	7.4	6.0	40.1	100.0
Rural	27.7	15.7	10.2	7.1	5.4	33.9	100.0
WEALTH QUINTILES							
Low	30.9	17.6	10.7	7.2	5.0	28.5	100.0
Low Medium	28.9	16.1	10.4	7.0	5.3	32.2	100.0
Medium	27.1	15.3	10.1	7.3	5.5	34.8	100.0
High Medium	24.7	14.1	10.2	7.3	5.7	37.9	100.0
High	21.7	12.9	9.8	7.5	5.9	42.3	100.0
